# Association between biophysical properties and anxiety in patients with sensitive skin

**DOI:** 10.1111/srt.13156

**Published:** 2022-04-13

**Authors:** Vildan Manav, Müge Göre Karaali, Ozan Erdem, Ayşe Esra Koku Aksu

**Affiliations:** ^1^ Department of Dermatology, University of Health Sciences İstanbul Training and Research Hospital Istanbul Turkey; ^2^ Master of Cosmetology İstanbul University Graduate School of Medicine İstanbul Turkey; ^3^ Department of Dermatology, Erzincan Binali Yildirim University Mengücek Gazi Training and Research Hospital Erzincan Turkey; ^4^ Deparment of Dermatology, University of Health Sciences Başakşehir Çam and Sakura City Hospital Istanbul Turkey

**Keywords:** anxiety, erythema, sebum, sensitive skin

## Abstract

**Background:**

Sensitive skin (SS) is a syndrome in which neurosensory disorders accompany epidermal barrier dysfunction. However, it is not yet clear how high anxiety levels affect the biophysical parameters of the skin in patients with SS.

**Objectives:**

We aimed to investigate the relationship between anxiety levels and facial neurosensitivity, the erythema index, sebum content, and sensitive skin scale scores in individuals with sensitive skin.

**Methods:**

The study was carried out on 35 individuals with SS and 40 without SS over three months. In the study, a questionnaire to detect the presence of sensitive skin, the sensitive skin scale for sensitive skin severity, the lactic acid sting test (LAST) to show facial neurosensitivity, a Mexameter for erythema index measurement, and a Sebumeter for sebum content measurement were used. In addition, the anxiety levels of the patient and control groups were measured using the hospital anxiety and depression scale (HADS).

**Results:**

While the HADS‐Anxiety scores were found to be significantly higher in patients with sensitive skin, there was no significant difference in the HADS‐Depression scores. Moreover, a strong positive correlation was found between the HADS‐Anxiety scores and the erythema index in patients with sensitive skin.

**Conclusions:**

Sensitive skin is a disorder that can sometimes occur without any dermatological examination findings. In particular, the sensations of the patients, along with their anxiety levels, are essential parameters that should be evaluated in the approach to patients with sensitive skin.

## 1 INTRODUCTION

“Sensitive skin (SS)” is defined by a special interest group, the International Forum for the Study of Itch1,^1^ as skin that develops an uncomfortable sensation in reaction to stimuli that skin should not normally react to various pathophysiological mechanisms are thought to be implicated in sensitive skin development.^2^ Neuropathy‐like discomfort sensations, such as burning, stinging, pain, and itching, are characteristic features of sensitive skin; therefore, it is postulated that a neurosensory disorder accompanied by epidermal barrier dysfunction plays a significant role in the pathogenesis of SS.^3^, ^4^

Psychological stressors may activate the hypothalamic/pituitary/adrenal axis and lead to the secretion of stress hormones. It has been shown that these secreted stress hormones may cause epidermal barrier dysfunction by decreasing stratum corneum (SC) hydration, increasing transepidermal water loss, and altering epidermal lipids and structural proteins.^3^ These epidermal alterations significantly affect the skin's barrier function, which is the primary topic of debate for developing sensitive skin.^4^ Another proposed underlying pathogenic factor for SS development is the presence of small fiber neuropathy.^5^ The association between increased anxiety levels and small fiber neuropathy is well established in fibromyalgia, diabetic neuropathy, and burning mouth syndrome.^6^, ^7^, ^8^, ^9^, ^10^ The role of depression and psychiatric factors in the development of sensitive skin has also been shown in the previous studies.^11^, ^12^, ^13^ However, the pathogenic role of anxiety in patients with SS has not been well studied in the literature. In the present study, we aimed to evaluate facial skin neurosensitivity via the lactic acid sting test (LAST), erythema index (EI), skin sebum levels, and sensitive skin scale (SSS) and reveal its association with anxiety levels in patients with sensitive skin.

## 2 METHODS

### 2.1 Study design and participants

We designed a prospective clinical study that collected data from September to December 2020. Seventy‐five participants aged 18−64 years were enrolled in the study after completing a self‐assessment questionnaire concerning facial skin neurosensitivity.^14^ In addition, all participants completed a written consent form before the study. Ethics committee approval was received from the Tertiary Hospital Scientific Research and Publication Ethics Board (protocol number: 2487, date: 07.24.2020).

Participants were selected using a quota method (age and gender) by a healthcare professional in our clinic. Before the measurements, the participants were asked to complete the self‐assessment questionnaire, the SSS, and the hospital anxiety and depression scale (HADS). All measurements tested in the face area were made by a single dermatologist who was blind to the questionnaire and scale results. Patients with systemic or autoimmune diseases, those with a personal or familial history of atopy or allergy, those under systemic or topical medication (glucocorticosteroid, acne, antibiotic), those with active lesions on the face, and those who were pregnant or lactating were excluded from the study.

### 2.2 The self‐assessment questionnaire and sensitive skin scale (SSS)^15^

A self‐assessment questionnaire was used to evaluate facial skin neurosensitivity. There were 13 questions on the questionnaire, and those who answered two of the first three positively were considered to have SS. Questions 4 to 7 assessed cosmetic sensitivity; those who answered three out of five positively were deemed to be sensitive to cosmetics. Questions 8 to 13 evaluated environmental sensitivity and those who answered three out of six positively were considered sensitive to the environment.

SSS was completed by participants who had SS and scored by quantifying ten symptoms (skin irritability, stinging, burning, sensation of heat, tautness, itching, pain, general discomfort, flushes, redness) from 0 to 10 via grading with a visual analog scale (0 = no feeling, 10 = very strong feeling).^16^ SS severity was then calculated by collecting all individual scores. The total score was in the range of 0−100.

### 2.3 Lactic acid sting test (LAST)

The LAST was performed using lactic acid (purity > 98%, The Pharmacy, Turkey), prepared at a concentration of 10% in distilled water. It was applied to the patients in their right nasolabial area using a cotton swab. Simultaneously, saline was applied to the contralateral area as a negative control at room temperature, as in a previous report.^17^ The stinging reaction was evaluated using a numerical scale (0 = no stinging, 1 = slight stinging, 2 = moderate stinging, 3 = severe stinging). The reaction scores were recorded at the initial phase and 2.5 minutes and 5 minutes after applying lactic acid. Cumulative scores ≥ 3 at 2.5 and 5 minutes were considered positive LAST results.

### 2.4 Measurement of the erythema index (EI) and skin sebum content

The EI and skin sebum content were measured with a Mexameter and Sebumeter (Courage & Khazaka Electronic GmbH, Cologne, Germany) in a closed environment with a constant temperature (21−23°C) and controlled air humidity (40%−60%). All participants sat quietly in the room for at least 30 minutes before measurement to acclimate their skin to the environmental conditions. Participants had been asked to discontinue all topical medications and cosmetics for the last 24 hours. In addition, they were asked to avoid skincare products, intense exercise, and sweating for at least 2 hours before the measurements.

A Mexameter 18 was used to measure erythema photometrically based on a remission principle. For each participant, the probe was held perpendicular to the forehead, nose, right and left nasolabial areas, and chin surface on the face, with no contact to the outer ring. Activating the light emitter resulted in the EI being captured by the Mexameter software; this result was in the range of 0−999 units. According to the following formula, the instrument analyzed the EI: El = 1,000 logs (at 655 nm, red light; 568 nm, green light).^18^ The results were expressed in arbitrary units (AU).

A Sebumeter SM 815 uses the difference in light intensity through a plastic tape cassette to indicate the amount of absorbed sebum. An opaque tape was pressed lightly on the face for 30 seconds to collect sebum. This study referred to the sebum content as the sebum level and expressed in μg/cm2. The measurements were performed on the forehead, right and left nasolabial areas, and chin.

### 2.5 The hospital anxiety and depression scale (HADS)

The HADS was used to measure symptoms of anxiety and depression. The HADS includes seven items assessing anxiety and seven items for depression, each with four possible answers. The lowest possible score for both subscales is 0, and the highest possible score is 21. Thus, the HADS‐Anxiety and HADS‐Depression scores are as follows: 0−7, normal; 8−10, borderline; and 11−21, need for treatment or further examination.

### 2.6 Statistical analysis

IBM SPSS Statistics version 24.0 software package (SPSS Inc., Chicago, IL, USA) was used for statistical analyses. Descriptive variables are presented as numbers and percentages for categorical variables; moreover, the mean, standard deviation, minimum, maximum, median, and interquartile range were used for numerical variables as appropriate. The categorical variables in the independent groups were compared using the Pearson Chi‐Square test. The normal distribution of the data was evaluated using the Shapiro–Wilk test. When the numerical variable fulfilled the normal distribution condition, comparisons of two independent groups were performed using the Student's *t*‐test. The Mann‐ Whitney U test was performed if the normal distribution condition was not provided. Relationships between numerical variables were determined using Spearman correlation analysis because parametric test conditions were not met. Two‐sided *p*‐values < 0.05 were considered statistically significant.

## 3 RESULTS

### 3.1 Demographic and clinical characteristics

Of the 75 participants, 40 (53.3%) were women, and 35 (46.7%) were men. The mean age of all participants included in the study was 34.8 ± 11.1 years. Having answered two of the first three questions of the questionnaire positively, 35 participants were classified as having SS. Among participants with SS, 17 (48.6%) were women and 18 (51.4%) were men; likewise, there were 23 (57.5%) female and 17 (42.5%) male participants without SS (*p* = 0.439). The mean age of participants with SS was 32.3 ± 11.8 years, whereas the mean age was 37±10.1 years in the participants without SS (*p* = 0.033). The Fitzpatrick skin phenotype did not significantly differ between the groups (*p* = 0.592). Thirty (85.7%) of 35 participants with SS had at least one concomitant dermatological disorder affecting the face. In contrast, only 17 (42.5%) participants without SS had an accompanying facial dermatological disorder (*p* < 0.001). The most common disorder was acne in both groups (25.7% in the SS group and 20% in the non‐SS group). However, only dermographism significantly differed between the groups (*p* = 0.003) (Table 1).

**TABLE 1:** Demographic and clinical characteristics of the participants

Characteristics	SS (n = 35)	Non‐SS (n = 40)	P‐value
Age in years, mean±sd	32.3±11.8	37±10.1	0.033 ^M^
Sex, n (%)			0.439^x2^
Female	17 (48.6)	23 (57.5)	
Male	18 (51.4)	17 (42.5)	
Fitzpatrick skin phenotype, n (%)			0.592^x2^
2	6 (17.1)	9 (22.5)	
3	19 (54.3)	17 (42.5)	
4	10 (28.6)	14 (35)	
Concomitant dermatologic disorders affecting face, n (%)	30 (85.7)	17 (42.5)	<0.001^x2^
Acne	9 (25.7)	8 (20)	0.555^x2^
Seborrheic dermatitis	7 (20)	3 (7.5)	0.174^F^
Dermographism	9 (25.1)	1 (2.5)	0.003^x2^
Rosacea	3 (8.6)	0 (0)	0.097^F^
Allergic contact dermatitis	1 (2.9)	2 (5)	0.999^F^
Psoriasis	0 (0)	2 (5)	0.495^F^
Urticaria	1 (2.9)	1 (2.5)	0.999^F^

SS, participants with sensitive skin; non‐SS, participants without sensitive skin; ^M^ Mann‐Whitney U test; ^x2^ Pearson Chi‐Square test; ^F^ Fisher's Exact test.

### 3.2 Questionnaire results and sensitive skin severity

**FIGURE 1:**
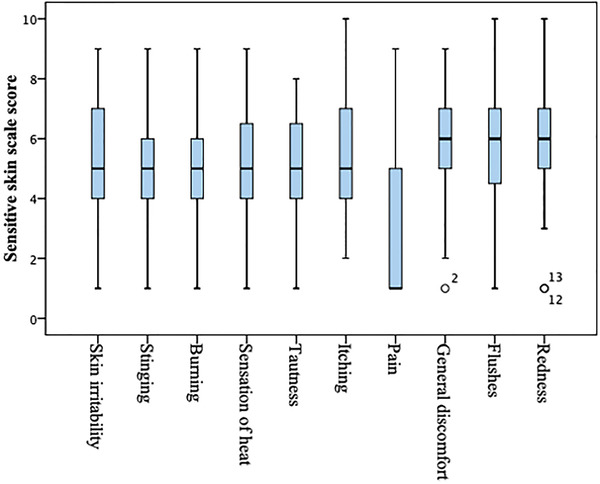
Sensitive skin scale in patients with sensitive skin. The mean sensitive skin severity score was calculated as 52.6 ± 13 out of 100

The self‐assessment questionnaire completed by the participants is presented in Table 2. According to questionnaire answers, among 35 participants who were defined as having SS, 12 (34.3%) also showed sensitivity to cosmetics. Moreover, 12 (34.3%) participants showed sensitivity to environmental factors. The results of the SSS, which participants with SS completed, are presented in Figure 1.

**TABLE 2:** The self‐assessment questionnaire

Questions	SS n (%)	Non‐SS n (%)	P‐value
1. Do you consider having sensitive facial skin?	34 (97.1)	12 (30.0)	<0.001
2. Do you think you have sensitive facial skin that is prone to irritation?	27 (77.1)	2 (5.0)	<0.001
3. Do you have “reactive” skin? (Do you experience stinging, burning, or itching sensations with and/or without associated redness.)	24 (68.6)	0 (0.0)	<0.001
4. Do you think the skin on your face is more sensitive than on other parts of your body?	14 (40.0)	2 (5.0)	<0.001
5. Does your skin react rapidly to cosmetics and toiletries?	12 (34.3)	2 (5.0)	0.001
6. Are there any cosmetic products that make your skin itch or cause a stinging or burning sensation?	14 (40.0)	2 (5.0)	<0.001
7. Have you ever experienced an adverse reaction to a cosmetic or toiletry product used on your skin?	12 (34.3)	2 (5.0)	0.001
8. Do you think your skin is especially sensitive to cold?	18 (51.4)	0 (0.0)	<0.001
9. Do you think your skin is especially sensitive to heat?	17 (48.6)	3 (7.5)	<0.001
10. Is your skin particularly sensitive to temperature change?	3 (8.6)	0 (0.0)	0.097
11. Does the wind tend to make your skin itchy or cause stinging or burning sensations?	14 (40.0)	0 (0.0)	<0.001
12. Does exposure to the sun makes your skin itchy or give rise to stinging or burning sensations?	20 (57.1)	2 (5.0)	<0.001
13. Does air pollution trigger a reaction in the skin on your face?	6 (17.1)	0 (0.0)	0.008

SS, participants with sensitive skin; non‐SS, participants without sensitive skin.

### 3.3 Biophysical properties

**FIGURE 2:**
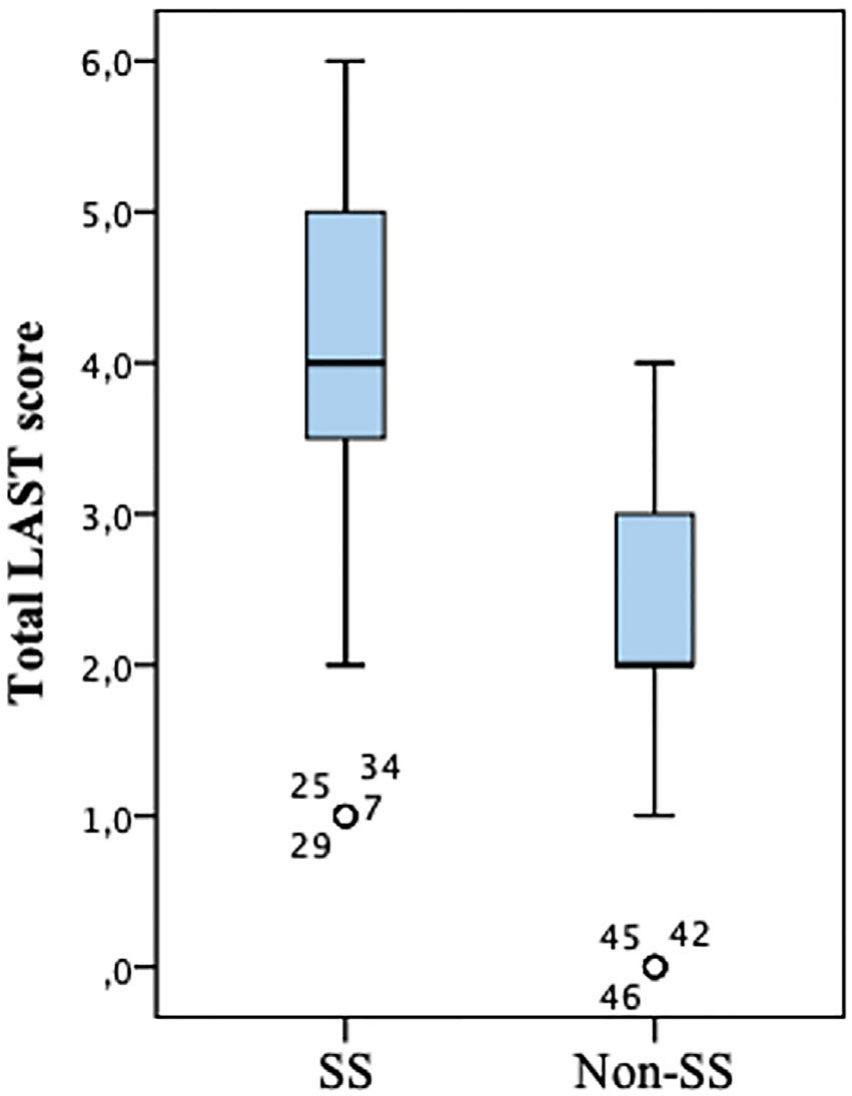
Distribution of LAST scores among subjects. LAST: Lactic acid stinging test; LAST positive: ≥ 3 and LAST negative: < 3; LAST positivity was statistically significantly higher in patients with sensitive skin (p < 0.001)

The mean total LAST score was 4.2 ± 1.5 in participants with SS and 2.1 ± 1.1 in participants without SS (Figure 2). A statistically significant difference was found in the baseline, 2.5‐minute, 5‐minute, and total LAST scores between participants with and without SS (for baseline LAST score, *p* = 0.001; for other LAST scores, *p* < 0.001). Thirty (85.7%) out of 35 participants with SS had a positive LAST (total last score ≥ 3), whereas only 12 (30%) out of 40 participants without SS showed a positive LAST (Table 3, *p* < 0.001).

**TABLE 3:** The biophysical properties of the participants

Biophysical properties	SS	Non‐SS	P‐value
Total LAST score, n (%)			<0.001^x2^
LAST negative: < 3	5 (14.3)	28 (70%)	
LAST positive: ≥ 3	30 (85.7)	12 (30%)	
Erythema index in AU, mean±sd			
Forehead	475.9±215.7	325.5±152.9	<0.001 ^M^
Right nasolabial fold	482±160	401±86.6	0.006 ^M^
Left nasolabial fold	480.5±143.1	398.8±113.8	0.004 ^M^
Nose	457.7±97	425.2±116.5	0.055 ^M^
Chin	466.8±76.1	477.1±156.4	0.318 ^M^
Mean	468.4±81.8	386.2±91.3	<0.001 ^M^
Sebum levels, μg/cm^2^			
Forehead	172.1±52.1	138.3±64.9	0.035 ^M^
Right nasolabial fold	121.4±59.5	83.3±53.9	0.010 ^M^
Left nasolabial fold	114.7±72.1	84.9±48.1	0.167 ^M^
Chin	98.3±63.8	54.8±44.6	0.001 ^M^
Mean	125.9±51.6	87±42.1	0.002 ^M^

^SS^ participants with sensitive skin; non‐SS, participants without sensitive skin; AU, arbitrary units.

^x2^Pearson Chi‐Square test.

^M^ Mann‐Whitney U test.

The EIs measured from the forehead, right and left nasolabial folds, nose, and chin are presented in Table 3. We observed significant differences between the groups in the forehead and right and left nasolabial folds (*p* < 0.001, *p *= 0.006, and *p *= 0.004, respectively). The mean EI was 468.4 ± 81.8 AU in participants with SS and 386.2 ± 91.3 AU in participants without SS (*p* < 0.001(Table 3).

Sebum levels showed significant differences between participants with SS and participants without SS in the forehead, right nasolabial fold, and chin (*p* = 0.035, *p* = 0.010, and *p* = 0.001, respectively). In addition, the mean sebum levels also differed, reaching 125.9 ± 51.6 AU in the SS group and 87.0 ± 42.2 AU in the non‐SS group (*p* = 0.002) (Table 3).

### 3.4 HADS‐anxiety and HADS‐depression scores

**FIGURE 3:**
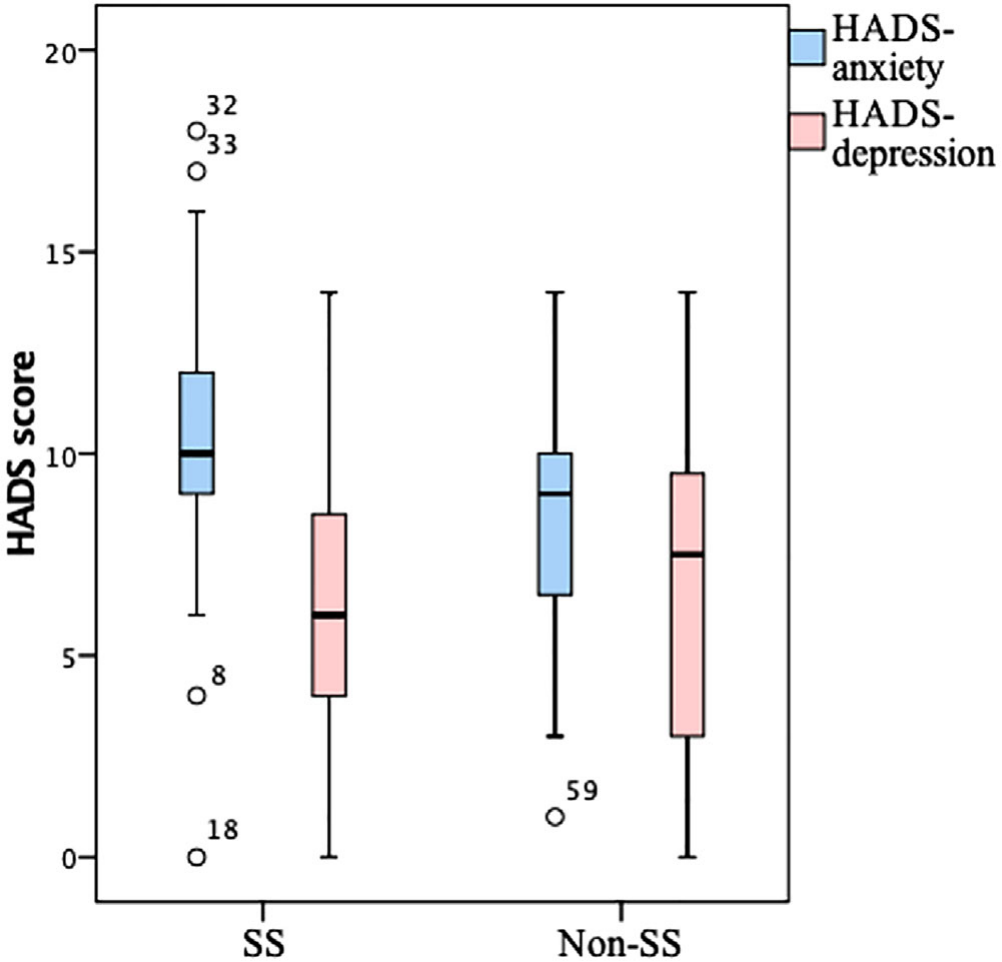
HADS‐Anxiety(HADSA) and Depression(HADSD) according to sensitivity. The mean HADS‐Anxiety scores were 10.3 ± 3.4 in participants with SS and 7.9 ± 3.0 in participants without SS . We found a statistically significant difference between the groups according to the HADS‐Anxiety scores (*p* = 0.001). In contrast, the mean HADS‐Depression scores were 6.5 ± 3.3 in participants with SS and 6.7 ± 3.7 in participants without SS. No differences were found between the groups in terms of the HADS‐Depression scores (*p* = 0.713)

The mean HADS‐Anxiety and HADS‐Depression scores of the participants are presented in Figure 3. Moreover, the number of participants who needed to be treated or have a further examination for anxiety (HADS **≥** 11) were higher in participants with SS compared with participants without SS (34.3% vs. 15.0%, respectively; *p* = 0.051).

### 3.5 Association between facial neurosensitivity measurements and HADS‐Anxiety and HADS‐Depression scores

**FIGURE 4:**
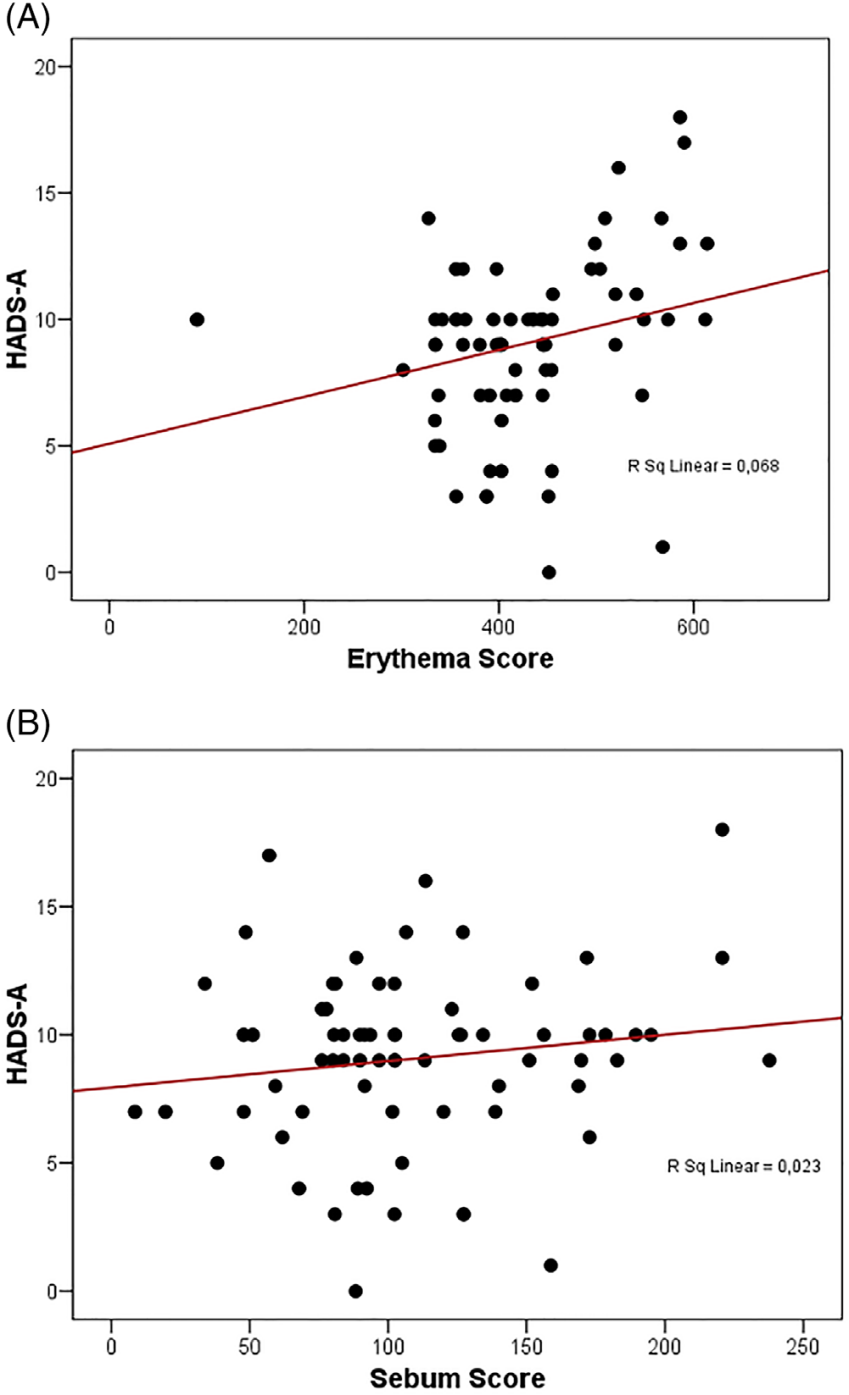
HADS‐Anxiety(HADSA)correlation between Erythema and Sebum

We further evaluated the association between the SSS, total LAST score, mean EI, mean sebum levels, and HADS‐Anxiety and HADS‐Depression scores in participants with SS (Table 4). The HADS‐Depression scores were not correlated with any of the facial neurosensitivity measurements. We found a strong positive correlation between the mean EI and HADS‐Anxiety scores (*r* = 0.678, *p* < 0.001); however, no further correlation was achieved according to the SSS score (*r* = 0.092, *p* = 0.599), total LAST score (*r* = −0.017, *p* = 0.921), and mean sebum level (*r* = 0.065, *p* = 0.709)(Figure 4).

**TABLE 4:** HADS‐Anxiety and HADS‐Depression scores according to Biophysical properties of patients with sensitive skin

	HADS‐anxiety scale score		HADS‐depression scale score	
Facial neurosensitivity measurements	r	p	r	p
Sensitive skin severity score	0.189	0.277	−0.086	0.622
Total LAST score	−0.017	0.921	0.062	0.725
Mean erythema index	0.678	<0.001	0.179	0.304
Mean sebum levels	0.065	0.709	−0.052	0.765

HADS, Hospital Anxiety and Depression Scale; LAST, lactic acid sting test. Spearman correlation was performed.

## 4 DISCUSSION

SS is an underestimated problem that dramatically affects the quality of life of affected people, and many internal and external factors influence the skin. Anxiety is one of the most important of these factors, and it may develop as a result of psychological stress. Many studies have already revealed the adverse effects of anxiety on the skin.^19^

Whereas depression scores were low in patients with SS, a significant relationship was observed between anxiety and SS in our study. Zafiriou et al. kept a connection between the Delusions–Symptoms–States Inventory/states of anxiety and depression subscale of anxiety, phobic anxiety, and patients with SS.^13^ In contrast, Misery et al. did not find a significant relationship between depressive symptoms and patients with SS.^11^

Moreover, in the study, the proportion of patients with SS requiring further examination and treatment (34.3%) because they had a high anxiety score(11‐21) was higher than the proportions of patients with psoriasis (22.7%), hand eczema (21.0%), atopic dermatitis (17.6%), leg ulcers (17.5%), and acne vulgaris (15.1%) requiring treatment for the same reason.^20^ Therefore, anxiety in SS patients should not be ignored, and relieving anxiety in these patients may reduce the level of feeling of disease symptoms.

Anxiety disrupts both the innate and adaptive immunity of the epidermis, disturbing the integrity of the SC and leading to skin barrier dysfunction.^21^ The mechanism of the effect of anxiety on the skin is not fully understood, but psychological stressors may activate the hypothalamic/pituitary/adrenal axis and lead to the secretion of stress hormones. Furthermore, it has been shown that these secreted stress hormones may cause epidermal barrier dysfunction by decreasing SC hydration, increasing transepidermal water loss, and altering epidermal lipids and structural proteins.^3^

Our study found that the significant increase in erythema and sebum levels in those with SS may be due to barrier dysfunction triggered by anxiety. Remarkably, a correlation was found between anxiety and EI but no correlation between sebum levels in patients with SS.

Erythema may indicate inflammation that develops due to processes caused by anxiety. In addition, as in the Study of Yosipovitch et al. on acne patients,^22^ it was observed in our study that neither psychological stress nor anxiety affected sebum levels or secretion.

In our study, we used LAST to assess hypersensitivity. We found significant positive results for hypersensitivity in sensitive skin. In addition, we found LAST positivity at a rate of 30% in those whose skin was not sensitive. However, Ding et al. stated that LAST could only be reliable in diagnosing sensitive skin with specific signs and symptoms such as tingling, itching, tightness, and scaling.^23^ However, there may be difficulties in diagnosing SS in patients where these findings are not evident. Interestingly, in this study, erythema levels were positively associated with anxiety in individuals with SS. Therefore, Erythema level measurement, a more rational method for detecting SS, can be considered.

There was a significant relationship between dermographism and SS in our study. It has been reported that such feelings as skin burning, tingling, and stinging seen in individuals with SS, together with dermographism, are also observed in electromagnetic hypersensitivity. From 18 epidemiological studies, *electromagnetic hypersensitivity* has been defined as neuropsychiatric effects caused by nonthermal microwave/lower frequency electromagnetic fields from cell/mobile phone base stations, excessive mobile phone usage, and wireless smart meters.^24^ Nowadays, as the use of these devices increases, SS can become even more remarkable.

### 4.1 Limitations

The most important limitation of our study is that we could not evaluate the indicators of impaired epidermal barrier function (increased transepidermal water loss, changes in skin surface lipids). In addition, the study was conducted during the coronavirus disease of 2019 (Covid‐19) pandemic, which affected the whole world; as a result, anxiety levels may already have been high. However, the results showed a statistically significant difference between the sensitive and the non‐sensitive groups regarding anxiety. Furthermore, our study shows that concomitant dermatological disorder affecting the face is higher in patients with sensitive skin. Therefore, it may cause anxiety scores to be higher in the sensitive skin group. Therefore, it would be more appropriate to conduct the study so that there is no significant difference between the control group and the sensitive skin in terms of concomitant dermatological disease.

## 5 CONCLUSION

Sensitive skin is a common condition but is often overlooked because of the subjective nature of its symptoms. It is essential to question the hypersensitivity of the skin caused by psychological stressor factors. Anxiety in SS patients should not be ignored, and relieving anxiety may reduce disease symptoms.

## CONFLICT OF INTEREST

The authors declare that there is no conflict of interest.
